# Molecular Features of SLC26A4 Common Variant p.L117F

**DOI:** 10.3390/jcm11195549

**Published:** 2022-09-22

**Authors:** Arnoldas Matulevičius, Emanuele Bernardinelli, Zippora Brownstein, Sebastian Roesch, Karen B. Avraham, Silvia Dossena

**Affiliations:** 1 Institute of Pharmacology and Toxicology, Paracelsus Medical University, 5020 Salzburg, Austria; 2 Department of Human Molecular Genetics & Biochemistry, Faculty of Medicine and Sagol School of Neuroscience, Tel Aviv University, Tel Aviv 6997801, Israel; 3 Department of Otorhinolaryngology, Head and Neck Surgery, Paracelsus Medical University, 5020 Salzburg, Austria

**Keywords:** hearing loss, *SLC26A4*, pendrin, DFNB4, Pendred syndrome, functional testing

## Abstract

The *SLC26A4* gene, which encodes the anion exchanger pendrin, is involved in determining syndromic (Pendred syndrome) and non-syndromic (DFNB4) autosomal recessive hearing loss. *SLC26A4* c.349C>T, p.L117F is a relatively common allele in the Ashkenazi Jewish community, where its minor allele frequency is increased compared to other populations. Although segregation and allelic data support the pathogenicity of this variant, former functional tests showed characteristics that were indistinguishable from those of the wild-type protein. Here, we applied a triad of cell-based assays, i.e., measurement of the ion transport activity by a fluorometric method, determination of the subcellular localization by confocal microscopy, and assessment of protein expression levels, to conclusively assign or exclude the pathogenicity of SLC26A4 p.L117F. This protein variant showed a moderate, but significant, reduction in ion transport function, a partial retention in the endoplasmic reticulum, and a strong reduction in expression levels as a consequence of an accelerated degradation by the Ubiquitin Proteasome System, all supporting pathogenicity. The functional and molecular features of human pendrin p.L117F were recapitulated by the mouse ortholog, thus indicating that a mouse carrying this variant might represent a good model of Pendred syndrome/DFNB4.

## 1. Introduction

Pendrin (SLC26A4) [1] is a multifunctional exchanger for monovalent anions, including I^−^, Cl^−^, HCO_3_^−^, OH^−^, SCN^−^, and formate [2], and is abundantly expressed in the inner ear [3], thyroid [1], and kidney [4]. In the cochlea, endolymphatic sac and duct, and vestibular system of the inner ear, pendrin is expressed on the apical membrane of epithelial cells and controls the ion composition, pH, and volume of the endolymph, most likely by secreting HCO_3_^−^ and reabsorbing Cl^−^ ions [5,6]. In the thyroid, pendrin is expressed on the apical membrane of thyrocytes and participates in the transport of iodide into the colloid, which is an essential step in the synthesis of thyroid hormones [7]. In the kidney, pendrin is expressed on the apical membrane of beta and non-alpha, non-beta intercalated cells of the cortical collecting duct and connecting tubule, and plays a major role in the control of systemic pH and blood pressure [8].

Pathogenic variants of the pendrin protein rising from sequence alterations in the *SLC26A4* gene (OMIM *605646) are involved in determining two of the most common forms of genetically inherited hearing loss, i.e., Pendred syndrome (phenotype MIM number 274600) and non-syndromic autosomal recessive deafness B4 (DFNB4, phenotype MIM number 600791) [9]. Children with Pendred syndrome/DFNB4 are often born with residual hearing, which is lost around the time of speech acquisition, with profound impact on language onset and speech discrimination. On the other hand, several patients manifest either fluctuating and progressive hearing loss or sudden hearing loss in the adult life [10]. In most cases, deafness is associated with malformations of the inner ear, i.e., an enlarged vestibular aqueduct (EVA) with or without cochlear incomplete partition II (Mondini cochlea) and possibly vestibular dysfunction [11]. However, there are reports of patients with biallelic *SLC26A4* pathogenic variants in whom no EVA could be observed [12]. Furthermore, the same pathogenic variant might be involved in different phenotypes in different patients, ranging from EVA to Pendred syndrome (i.e., p.Val138Phe, p.Gly209Val, p.Leu236Pro, p.Glu384Gly, and p.Leu445Trp) (reviewed in [13]).

In Pendred syndrome, patients also suffer from thyroid dysfunction consisting in a partial iodide organification defect disclosed by a positive perchlorate discharge test, with incompletely penetrant goiter appearing around puberty [14].

Discrimination between pathogenic and non-pathogenic gene variants is a major issue in gene diagnostic and is of the utmost importance for assisting patients with an accurate diagnosis, prognosis, genetic counseling, and planning of an appropriate intervention. Up to now, 1029 sequence alterations of the *SLC26A4* gene have been reported (https://www.ncbi.nlm.nih.gov/clinvar, accessed on the 13 June 2022) and only in part characterized concerning their clinical significance. Three hundred and twenty-eight of them are missense mutations generating protein variants, of which 167 have been classified as pathogenic/likely pathogenic. Pathogenic protein variants derived from nonsense mutations, altered splicing, and frameshift have also been described. The American College of Medical Genetics and Genomics (ACMG) and the Association for Molecular Pathology (AMP) recommended categorizing gene variants identified in Mendelian disorders as ‘pathogenic’, ‘likely pathogenic’, ‘uncertain significance’, ‘likely benign’, and ‘benign’ [15]. The Hearing Loss Variant Curation Expert Panel adapted the ACMG/AMP guidelines for the interpretation of sequence variants in hearing loss genes [16]. According to these recommendations, minor allele frequency (MAF), computational analysis, functional data, segregation data, de novo occurrence, allelic configuration (in cis or in trans with an established pathogenic variant), and patient’s phenotype highly consistent with a specific hearing loss syndrome should be taken into account when interpreting the clinical significance of a given variant. However, a number of *SLC26A4* variants remain controversial regarding their possible pathogenic potential.

Functional testing can represent a strong criterion for pathogenicity assignment or exclusion and, concerning pendrin variants, was made by the quantification of radiolabeled chloride or iodide fluxes in various heterologous expression systems, including *Xenopus laevis* oocytes, insect Sf9 cells, mammalian and human cells [17,18,19,20,21,22], or even in primary cultures of thyrocytes from a patient with Pendred syndrome [23]. In addition, fluorometric methods measuring intracellular pH variations or halide fluxes were developed [24,25,26]. Testing of the function of pendrin variants found in deaf patients revealed a partial or total impairment of pendrin ion transport activity [18]. The main pathomechanism of disease was believed to be the retention of the misfolded pendrin protein variants within subcellular compartments [27]. Recently, however, we showed that the reduction of protein levels, rather than mis-localization, is the key feature of pathogenic pendrin protein variants [28,29].

Here, we exploited a triad of cell-based assays, specifically the determination of ion transport function, subcellular localization, and protein abundance, to conclusively assign or exclude the pathogenicity of the *SLC26A4* variant NM_000441.1:c.349C>T, p.Leu117Phe (p.L117F), dbSNP: rs145254330, a founder variant of the Ashkenazi Jewish population with controversial findings regarding its pathogenicity [30]. Our assays supported the pathogenic potential of this variant and further underscored that the determination of protein levels can disclose molecular defects in the face of mild impairments in ion transport activity.

## 2. Materials and Methods

### 2.1. Cell Culture

Human embryonic kidney (HEK) 293 Phoenix [31] and HeLa cells (human cervical adenocarcinoma, CCL-2, directly obtained from American Type Cell Culture Collection (ATCC), Manassas, VA, USA) were cultured in Minimum Essential Eagle Medium (Sigma-Aldrich, St. Louis, MO, USA) supplemented with 10% fetal bovine serum (GIBCO, Thermo Fisher, Waltham, MA, USA), 2 mM L-glutamine, 100 U/mL penicillin, 100 mg/mL streptomycin, and 1 mM pyruvic acid (sodium salt). The cells were maintained at 37 °C, 5% CO_2_, 95% air, and 100% humidity. Subcultures were routinely established every second to third day by seeding the cells into 100 mm diameter Petri dishes following trypsin/ethylenediaminetetraacetic acid (EDTA) treatment.

For Western blotting and confocal imaging, cells were seeded into six-well plates, grown overnight to approximately 50% confluence, and transiently transfected with 3 μg plasmid DNA by the calcium phosphate co-precipitation method (HEK 293 Phoenix) or 1.5 μg of plasmid DNA and 3 μL METAFECTENE PRO^®^ (Biontex, Munich, Germany), following the manufacturer’s instructions (HeLa). Medium was replaced 6–8 h after transfection, and cells were processed 48 (HEK 293 Phoenix) or 72 h (HeLa) after transfection.

### 2.2. Plasmid Constructs

The pTARGET (Promega Corporation, Madison, WI, USA) vector containing the open reading frame (ORF) coding for wild type human SLC26A4 (NCBI Sequence ID: NM_000441.2) or *Mus musculus* SLC26A4 (NCBI Sequence ID: NM_011867.4) with or without a hexahistidine tag at the C-terminus was used for functional testing and Western blot.

For the colocalization and determination of pendrin expression levels via imaging, the pEYFPN1 vector (Clontech Laboratories Inc., Mountain View, CA, USA) was used. In this vector, the ORF of human or mouse pendrin was subcloned in frame with the ORF of the enhanced yellow fluorescent protein (EYFP). After the transfection of these constructs in cells, pendrin was produced with the EYFP fused to its C-terminus.

The c.349C>T pendrin sequence alteration leading to the p.L117F protein variant was made using the QuikChange^®^ site-directed mutagenesis kit (Stratagene) according to the manufacturer’s protocol using the following primers: fwd, 5′ CCTGTCGGATATGGTTTCTACTCTGCTTTTTTCCC 3′, and rev, 5′ GGGAAAAAAGCAGAGTAGAAACCATATCCGACAGG 3′ for human pendrin and fwd, 5′ CCTGTTCAGTTCGGTTTCTACTCTGCCTTTTTCCC 3′, and rev, 5´ GGGAAAAAGGCAGAGTAGAAACCGAACTGAACAGG 3′ for mouse pendrin. All plasmid inserts were sequenced before use in experiments (Microsynth AG, Balgach, Switzerland).

### 2.3. Functional Testing

The ion transport activity of pendrin was determined by measuring the iodide influx in pendrin-transfected cells by a fluorometric method, as formerly described [24,28,29,32,33,34,35,36,37,38]. Shortly, cells were seeded into black 96-well plates, grown overnight, and co-transfected with 0.12 μg/well of a plasmid encoding for the iodide-sensitive EYFP variant H148Q;I152L [39] and 0.12 μg/well of a pTARGET plasmid encoding for pendrin. Control cells were co-transfected with EYFP H148Q;I152L and the empty pTARGET vector. Transfection was performed by the calcium phosphate co-precipitation method.

Experiments were performed at room temperature 48 h post-transfection. Cells were initially washed and bathed in 70 μL of a high-chloride solution (in mM: KCl 2, NaCl 135, CaCl_2_ 1, MgCl_2_ 1, D-glucose 10, 4-(2-hydroxyethyl)-1-piperazineethane sulfonic acid (HEPES) 20, 308 mOsm/KgH_2_O adjusted with mannitol, pH 7.4), and the baseline fluorescence intensity was measured (1 measurement/sec for 3 sec). Subsequently, 140 μL of a high-iodide solution (in mM: KCl 2, NaI 135, CaCl_2_ 1, MgCl_2_ 1, D-glucose 10, HEPES 20, 308 mOsm/KgH_2_O adjusted with mannitol, pH 7.4) were injected into each well, and the fluorescence intensity was followed for 16 sec. Fluorescence intensity was quantified with the VICTOR^TM^ X3 Multilabel Plate Reader (Perkin Elmer, Waltham, MA, USA) equipped with a liquid dispenser and the following filters: excitation: F485 (excitation center wavelength (CWL): 485 nm, bandwidth: 14 nm), emission: F535 (emission CWL: 535 nm, bandwidth: 25 nm).

The background fluorescence measured in cells transfected with 0.24 μg of the pTARGET vector with no EYFP H148Q;I152L was subtracted from all of the other fluorescence measurements of the same 96-well plate. Data were expressed as % fluorescence variations, and a negative fluorescence variation indicated a flux of iodide from the extracellular milieu to the intracellular environment.

### 2.4. Determination of Protein Expression Levels by Quantitative Imaging

Quantitative imaging was performed as formerly described [28,29]. Shortly, cells expressing the fusion proteins SLC26A4-EYFP or SLC26A4-EYFP were fixed with 3% paraformaldehyde for 30 min, counterstained with 0.1 μg/mL 4′,6-diamidino-2-phenylindole (DAPI) for 10 min, thoroughly washed, and imaged in Hank’s balanced salt solution (HBSS, Sigma-Aldrich). Imaging was performed with a Leica TCS SP5II AOBS confocal microscope (Leica Microsystems, Wetzlar, Germany) equipped with a HCX PL APO 63×/1.20 Lambda blue water immersion objective and controlled by the LAS AF SP5 software version 2.7.3.9723 (Leica Microsystems). EYFP was excited with the 514 nm line of the Argon laser, and emission was detected between 525 and 600 nm; DAPI was excited with a diode laser (405 nm), and emission was detected between 430 and 470 nm. Laser power and photomultipliers gain were kept rigorously constant for the acquisition of all images. To obtain pendrin expression levels normalized for the cell density, the fluorescence intensity (in average levels of gray) of the whole imaging field in the EYFP emission window was subtracted for the background fluorescence and normalized for the background-subtracted fluorescence intensity in the DAPI emission window.

### 2.5. Colocalization

Colocalization of SLC26A4-EYFP with subcellular markers was determined as previously described [28]. Shortly, to stain the plasma membrane, living cells were washed three times with ice-cold HBSS, incubated on ice for 5 min with 1.25 μg/mL CellMask^TM^ Deep Red plasma membrane stain (C10046, Invitrogen Molecular Probes, Thermo Fisher, Waltham, MA, USA) in HBSS, washed again three times with ice-cold HBSS, and imaged immediately. To stain the endoplasmic reticulum, living cells were washed three times with room temperature HBSS, incubated for 20 min at 37 °C and 5% CO_2_ with 1 μM ER-Tracker^TM^ Red (glibenclamide BODIPY^®^TR, E34250, Invitrogen Molecular Probes) in Krebs-Henseleit buffer (pH 7.4, Sigma-Aldrich), washed again three times, and immediately imaged in HBSS at room temperature.

EYFP was excited with the 514 nm line of the Argon laser, and emission was detected in the 525–600 or 525–555 nm range for colocalization with the plasma membrane or the endoplasmic reticulum, respectively. CellMask^TM^ Deep Red was excited at 633 nm with a HeNe laser, and emission was detected in the 655–750 nm range. ER-Tracker^TM^ Red was excited at 561 nm with a diode-pumped solid-state (DPSS) laser, and emission was detected in the 590–670 nm range.

Imaging was performed by sequential acquisition with a Leica TCS SP5II AOBS confocal microscope (Leica Microsystems). Colocalization was quantified within regions of interest comprising single transfected cells and expressed as the Pearson’s correlation coefficient [40], overlap coefficient, and colocalization rate with the Colocalization tool of the LAS AF SP5 software (Leica Microsystems).

### 2.6. Western Blotting

Cells were collected by centrifugation, and the cell pellet was washed in ice-cold phosphate buffered saline (PBS). Subsequently, cells were lysed in RIPA buffer (Sigma-Aldrich), the cellular debris was discarded by centrifugation at 900× *g* for 10 min at 4 °C, the supernatant was collected, and the protein content was quantified with the DC^TM^ Protein Assay (Bio-Rad, Hercules, CA, USA). Typically, 20 μg of total proteins were solubilized in a 4× loading dye containing 100 mM dithiothreitol and 8% SDS, separated on a 7.5% polyacrylamide gel containing 0.1% SDS, and electroblotted on a polyvinylidene fluoride (PVDF) membrane (Bio-Rad, Basel, Switzerland) by applying a constant voltage (75 V) for 2 h at 4 °C. PVDF membranes were blocked for 1 h at room temperature in 5% non-fat dry milk diluted in Tris-buffered saline (150 mM NaCl and 15 mM Tris-HCl) containing 0.1% Tween 20 (TBST) and incubated overnight at 4 °C with a rabbit anti-pendrin polyclonal antibody raised against amino acids 586–780 of human pendrin (sc-50346, Santa Cruz Biotechnology, Dallas, TX, USA) diluted 1:500. The housekeeping protein calreticulin/calregulin was detected with a mouse monoclonal antibody (sc-373863, Santa Cruz Biotechnology) diluted 1:100. Membranes were washed three times in TBST and incubated for 1 h at room temperature, with the secondary antibodies diluted 1:20,000 in PBS and 5% non-fat dry milk (goat anti-rabbit or goat anti-mouse IRD-800 CW, both from LI-COR, Lincoln, NE, USA). The signal of immunocomplexes was revealed using the Odyssey (LI-COR) infrared imaging system. Densitometric analysis was performed using the ImageJ (1.49v) software.

### 2.7. Real-Time qPCR

Extraction of total RNA was performed with the RNeasy^®^ Micro Kit (Qiagen, Hilden, Germany). A total of 1 μg total RNA was used for the reverse transcription reaction with the QuantiTect^®^ reverse transcription kit for cDNA synthesis with integrated removal of genomic DNA contamination (Qiagen). qPCR reactions contained 5 µL of cDNA template in a 20 µL final volume with 1× PrimeTime^®^ qPCR primers (Integrated DNA Technologies, Coralville, IA, USA) and 1× GoTaq^®^ qPCR Master Mix (Promega). Real-time PCR of all samples including a minus reverse transcriptase control and no template control was performed in technical duplicates on the Rotor Gene Q instrument (Qiagene). mRNA levels were normalized for those of the housekeeping *POLR2A* transcript. Total RNA from a human thyroid (Cat. No. 636536) was from Clonthech.

### 2.8. Bioinformatics

Protein or DNA sequence alignments were made with the 13.0.7 version of MacVector (MacVector Inc., Apex, NC, USA).

### 2.9. Salt and Chemicals

All salt and chemicals were of *pro analysis* grade. Carfilzomib (PR-171) was from Selleckchem (Planegg, Germany). Carfilzomib stock solutions were prepared in dimethylsulfoxide (DMSO) (Sigma-Aldrich) and stored at −80 °C.

### 2.10. Statistical Analysis

All data were expressed as arithmetic means ± standard error of the mean (S.E.M.). For statistical analysis and the generation of graphics, GraphPad Prism (version 5.0b for Mac OS, GraphPad Software, San Diego, CA, USA) and Excel (Microsoft, Redmond, WA, USA) software were used. Significant differences between data sets were determined by the unpaired Student’s *t*-test or ANOVA with Bonferroni’s ad hoc post-test, as appropriate. Statistically significant differences were assumed at *p* < 0.05; (*n*) corresponds to the number of independent measurements.

## 3. Results

### 3.1. The Ion Transport Activity of Pendrin p.L117F Is Moderately but Significantly Reduced Compared to the Wild Type

**Figure 1:**
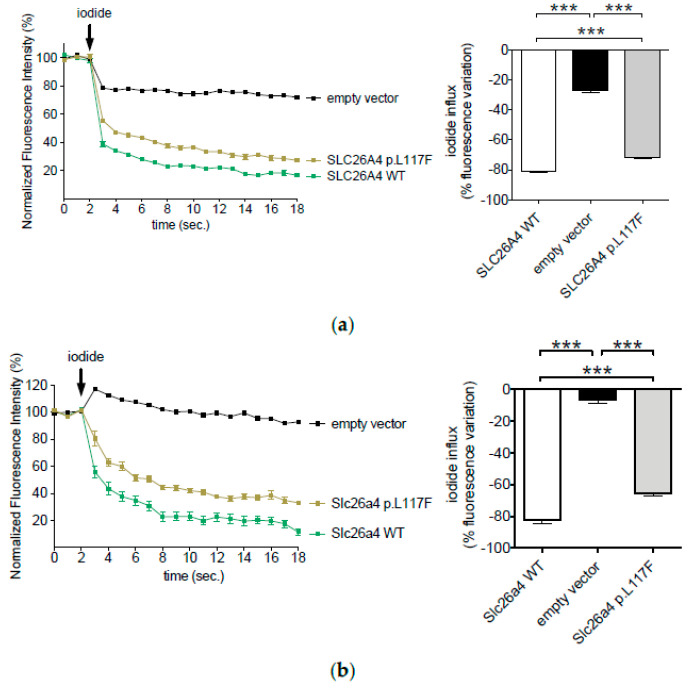
Ion transport activity of human (**a**) and *Mus musculus* (**b**) wild-type pendrin and pendrin variant p.L117F. HEK 293 Phoenix cells were co-transfected with wild-type (WT) or p.L117F pendrin, and the iodide sensor enhanced yellow fluorescent protein (EYFP) H148Q;I152L or EYFP H148Q;I152L alone (empty vector) and bathed in chloride- or iodide-containing solutions. The arrow indicates the addition of the iodide-containing solution to the bath. Left panels show the average fluorescence intensity over time normalized for the average fluorescence intensity measured in the chloride-containing solution. Right panels represent the % decrease in fluorescence intensity determined over the experimental period (19 s). *n* = 60 measurements from 5 independent experiments. *** *p* < 0.0001, one-way ANOVA with Bonferroni’s multiple comparison post-test.

Human pendrin or its mouse ortholog were transiently expressed in HEK 293 Phoenix cells, and their ion transport activity was measured with a fluorometric method. For this, cells were co-transfected with the iodide-sensitive EYFP H148Q;I152L (see Methods), and cells expressing EYFP H148Q;I152L but no pendrin (empty vector) served to assess the endogenous ion transport in these cells. Following the exposure of cells to an extracellular solution containing iodide, an intracellular fluorescence decrease indicated an influx of this ion towards the cytosol. Cells expressing human or mouse pendrin exhibited a similar fluorescence decrease of approximately 80% over the experimental period, and this decrease was significantly higher compared to that measured in cells expressing no pendrin. This behavior is consistent with the I^−^/Cl^−^ anion exchange activity of pendrin and is in good agreement with former observations [24,28,29,32,33,34,35,36,37,38]. Cells expressing human or mouse pendrin p.L117F still exhibited a substantial I^−^/Cl^−^ anion exchange activity, which was, however, reduced compared to the wild type. The reduction of function of human (17%) and mouse (22%) pendrin appeared to be moderate, but it was statistically significant (Figure 1).

### 3.2. The Protein Expression of Pendrin p.L117F Is Substantially Reduced Compared to the Wild Type

**Figure 2:**
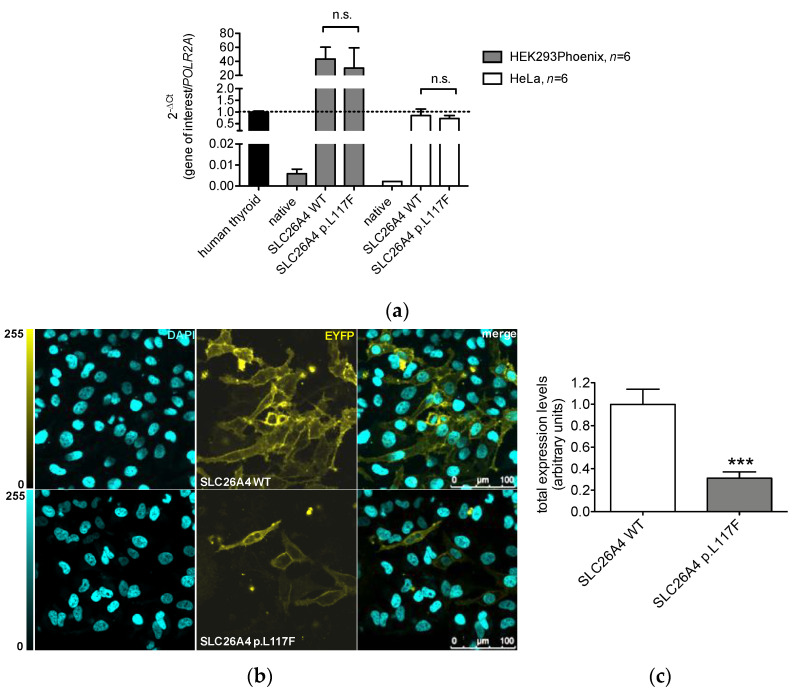

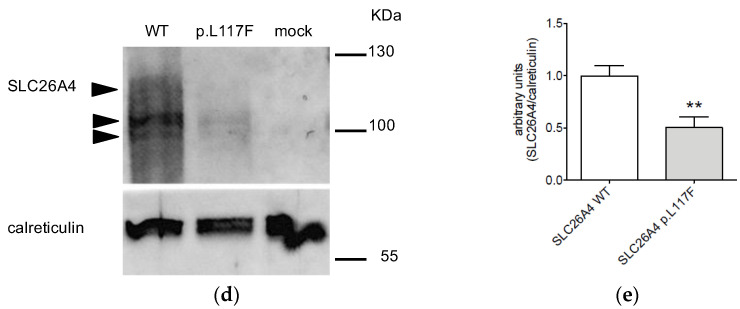
Transcript and protein levels of human wild type pendrin and pendrin variant p.L117F. (**a**) HEK 293 Phoenix or HeLa cells were left untransfected (native) or transfected with the same plasmid constructs used for the determination of protein levels, and the *SLC26A4* transcript levels were quantified by qPCR. Human thyroid was tested in parallel as a reference. *n* refers to the number of biological replicates. (**b**) Representative images of fixed Hela cells transfected with wild type (WT) or p.L117F SLC26A4-EYFP (yellow) and counterstained with DAPI (cyan). The corresponding merge images are shown. Scale bar: 100 μm. (**c**) Pendrin protein expression levels expressed as fluorescence intensity (levels of gray) normalized for the cell density. *n* = 24 imaging fields from 4 independent subcultures. (**d**) Representative western blot on HEK 293 Phoenix cells transfected with wild type (WT) or p.L117F SLC26A4 or with an empty vector (mock). (**e**) Densitometry of *n* = 6 samples from 3 independent subcultures. n.s., not significant, ** *p* < 0.01, *** *p* < 0.0001, unpaired, two-tailed Student´s *t* test.

Human pendrin was transiently expressed in HEK 293 Phoenix or HeLa cells, and the corresponding transcript and protein levels were measured by qPCR, quantitative imaging, and western blot. Transcript levels in transfected HeLa cells reached those of human thyroid and increased approximately 40-fold in HEK 293 Phoenix cells. No significant difference between the wild type and p.L117F transcript levels were detected in either cell lines (Figure 2a).

Protein levels were determined in HeLa cells by quantitative imaging. Compared to the wild type, cells expressing SLC26A4 p.L117F were less numerous and had a weaker signal, with occasional cells showing the retention of protein in the intracellular compartments (Figure 2b). Quantitative analysis showed a significant (69%) reduction of expression of SLC26A4 p.L117F compared to the wild type (Figure 2c). These findings were reproduced in HEK 293 Phoenix cells, where western blot followed by densitometry showed a 50% reduction of expression of SLC26A4 p.L117F compared to the wild type (Figure 2d,e).

### 3.3. Pendrin p.L117F Is Mostly but Not Exclusively Localized at the Plasma Membrane

**Figure 3:**
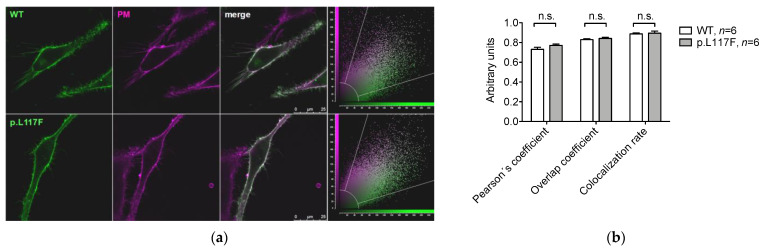
Colocalization of human wild-type pendrin and pendrin variant p.L117F with the plasma membrane. (**a**) Representative images of living Hela cells transfected with wild type (WT) or p.L117F SLC26A4-EYFP (green) and stained with the plasma membrane (PM) marker CellMask^TM^ Deep Red (magenta). The corresponding merge images and scatter plots are shown. Scale bar: 25 μm. (**b**) Average Pearson’s correlation coefficient, overlap coefficient, and colocalization rate. *n* refers to the number of cells from 3 independent subcultures. n.s., not significant, unpaired, two-tailed Student´s *t* test.

**Figure 4:**
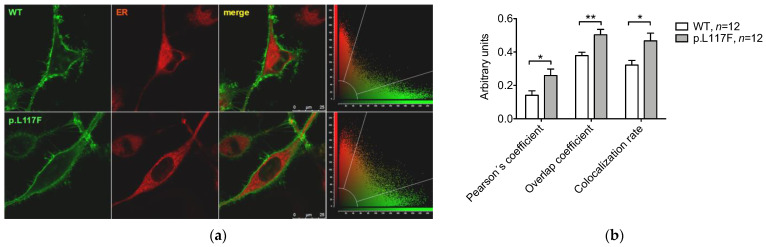
Colocalization of human wild-type pendrin and pendrin variant p.L117F with the endoplasmic reticulum. (**a**) Representative images of living Hela cells transfected with wild type (WT) or p.L117F SLC26A4-EYFP (green) and stained with the endoplasmic reticulum (ER) marker ER-Tracker^TM^ Red (red). The corresponding merge images and scatter plots are shown. Scale bar: 25 μm. (**b**) Average Pearson´s correlation coefficient, overlap coefficient, and colocalization rate. *n* refers to the number of cells from 3 independent subcultures. * *p* < 0.05, ** *p* < 0.01, unpaired, two-tailed Student´s *t* test.

Human wild type pendrin and pendrin p.L117F were expressed in HeLa cells, and their subcellular localization was determined by colocalization with markers of the plasma membrane and the endoplasmic reticulum. Pendrin p.L117F appeared to correctly target the plasma membrane (Figure 3a), and, accordingly, the corresponding colocalization parameters failed to reveal a significant difference with the wild type (Figure 3b). However, the Pearson´s colocalization coefficient, overlap coefficient, and colocalization rate of pendrin p.L117F and the endoplasmic reticulum statistically increased compared to those of the wild type, thus revealing a partial but significant retention of pendrin p.L117F in this subcellular compartment (Figure 4).

### 3.4. Pendrin p.L117F Is Degraded by the Ubiquitin Proteasome System

**Figure 5:**
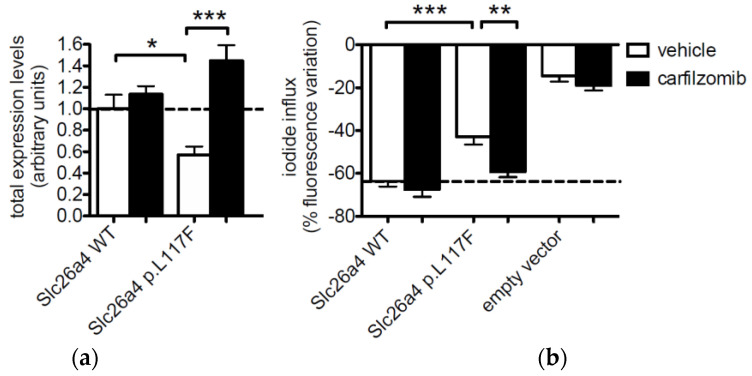
Expression and function of *Mus musculus* wild type pendrin and pendrin variant p.L117F in the presence of carfilzomib or its vehicle in HEK 293 Phoenix cells. (**a**) Cells were transfected with wild type (WT) or p.L117F SLC26A4-EYFP for 48 h and incubated with 1 μM carfilzomib or the vehicle (0.01% DMSO) for 16 h. Protein expression levels are expressed as fluorescence intensity (levels of gray) normalized for the cell density. *n* = 20 imaging fields from 4 independent subcultures. (**b**) Cells were co-transfected with wild type (WT) or p.L117F SLC26A4 and the iodide sensor enhanced yellow fluorescent protein (EYFP) H148Q;I152L or EYFP H148Q;I152L alone (empty vector) for 48 h and incubated with carfilzomib or the vehicle for 16 h. The % decrease in fluorescence intensity indicates an iodide influx from the bath solution towards the intracellular environment. *n* = 24 measurements from 4 independent experiments. * *p* < 0.05, ** *p* < 0.01, *** *p* < 0.001, one-way ANOVA with Bonferroni’s multiple comparison post-test.

The expression of *Mus musculus* pendrin in HEK 293 Phoenix cells denoted a significant (43%) reduction of expression of p.L117F compared to the wild type (Figure 5a), consistent with what was observed for the human ortholog. Following the exposure of cells to the Ubiquitin Proteasome System (UPS) inhibitor carfilzomib, a 2.5-fold increase in the expression of SLC26A4 p.L117F was observed. The increase in protein expression was paralleled by a significant increase in ion transport activity towards a level not significantly different from those of the wild type (Figure 5b). In contrast, the expression and function of wild-type pendrin were not significantly affected by carfilzomib. In addition, exposure to carfilzomib did not significantly change the endogenous ion transport activity in these cells.

## 4. Discussion

The *SLC26A4* variant NM_000441.1:c.349C>T, p.L117F was first described in the year 2000 by Reardon et al. in a patient of unspecified ethnicity with EVA and normal perchlorate discharge profile (an abnormal test was defined as a discharge of radio-iodide from the thyroid exceeding 10%). In this patient, the second pathogenic variant could not be detected [41]. Later, this variant was again found in a Caucasian patient with hearing loss and EVA in whom the second pathogenic variant was not identified [42]. These findings are not in conflict with a possible pathogenicity of p.L117F, as approximately 25% of patients with hearing loss and EVA lack identification of the second pathogenic allele in Caucasian cohorts [11].

Having an MAF of 0.0063 in a control population, this variant was initially categorized as benign based on the MAF threshold of 0.005 for autosomal recessive hearing loss variants [43].

Variant *SLC26A4* c.349C>T, p.L117F was also found in compound heterozygosis with the c.−4+1G>C variant in a patient with congenital deafness and EVA in a large multiethnic cohort including Caucasian, Hispanic, African American, Asian, Middle Eastern, Ashkenazi Jewish, mixed, and other ethnicity patients [44]. According to the ACMG/AMP guidelines, the detection of a variant in trans with another pathogenic variant supports pathogenicity for an autosomal recessive disease (pathogenic moderate criterion PM3) [15]. Based on these allelic data, segregation studies, as well as in silico predictions, this variant was classified as Likely Pathogenic in ClinVar (https://www.ncbi.nlm.nih.gov/clinvar/, accessed on 10 August 2022).

The allele frequency of *SLC26A4* c.349C>T, p.L117F is 0.013 in the Israeli Jewish deaf population and 0.007 in controls of the same ethnicity, compared to 0.0002 in other gnomAD populations. We recently reported the family pedigree and audiologic findings of two families of Ashkenazi Jewish ancestry with five children with hearing loss, of whom four are homozygotes for this variant. Homozygosity for *SLC26A4* c.349C>T, p.L117F has also been observed in four other Ashkenazi families during screening for childhood hearing loss by genetic clinics in Israel. Based on these considerations, the variant was recently reclassified to Pathogenic [16,30].

The expression of pendrin p.L117F with a C-terminal GFP tag in HeLa cells denoted a plasma membrane targeting similar to that of the wild type. In HEK 293 cells transiently co-transfected with the sodium-iodide symporter (NIS) SLC5A5, p.L117F exhibited a rate of iodide efflux statistically indistinguishable from that of the wild type [18]. In a study based on the stable doxycycline-dependent expression of pendrin missense variants in HEK293T-based cell lines, pendrin p.L117F showed normal Cl^−^/HCO_3_^−^ and Cl^−^/I^−^ exchange activity, localized at the plasma membrane and intracellular puncta, and did not affect gene splicing, thus showing functional and molecular features indistinguishable from those of the wild type [26].

**Figure 6:**
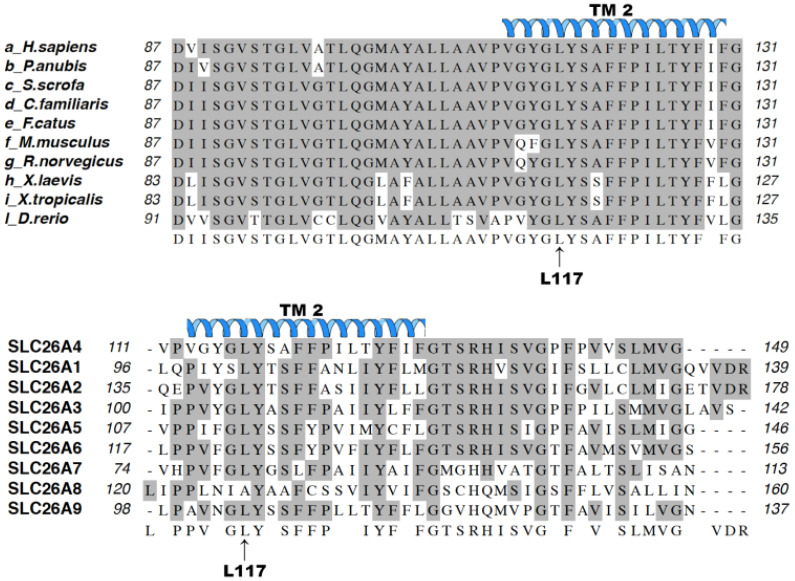
Multiple sequence alignment of the SLC26A4 protein sequence with its orthologues (top) and human paralogues (bottom). Amino acid residues conserved in all proteins are shaded in gray. The putative position of the second transmembrane (TM 2) α-helix according to [45] is indicated above the sequence. The position of the amino acid Leu117 (L117) is also given. (a) *H. sapiens* (NP_000432.1), (b) *P. Anubis* (XP_021791848.1), (c) *S. scrofa* (XP_003357559.1), (d) *C. familiaris* (XP_022260905.1), (e) *F. catus* (XP_003982698.1), (f) *M. musculus* (NP_035997.1), (g) *R. norvegicus* (NP_062087.1), (h) *X. laevis* (AAI69726.1), (i) *X. tropicalis* (NP_001107135.1), (l) *D. rerio* (NP_001159387.1), SCL26A4 (NP_000432.1), SLC26A1 (NP_998778.1), SLC26A2 (NP_000103.2), SLC26A3 (NP_000102.1), SLC26A5 (NP_945350.1), SLC26A6 (NP_075062.2), SLC26A7 (NP_001269285.1), SLC26A8 (NP_001180405.1), SLC26A9 (NP_443166.1).

Leucine (Leu) and Phenylalanine (Phe) are both neutral non-polar amino acids; however, the structural characteristics of the side chain, which is aliphatic for Leu and aromatic for Phe, appear different. This missense occurs in a residue that is completely conserved among pendrin orthologues from *H. sapiens* to *D. rerio* and highly conserved among paralogues (Figure 6) and, according to our structural model, falls within the second transmembrane helix of pendrin [45]. Therefore, it appeared surprising to us that the amino acid substitution did not affect the protein subcellular distribution and function. Thus, we applied three distinct molecular and functional cell-based assays to verify possible subtle alterations that may not have been disclosed by former studies.

The ion transport function of human pendrin appeared to be moderately (17%) but significantly reduced compared to the wild type (Figure 1a). Mild reductions in ion transport activity have been discussed as per se non-causative of hearing loss but possibly pathogenic in trans configuration with a different established pathogenic variant [21]. As a matter of fact, hearing loss patients homozygous for p.L117F have been described in the Ashkenazi Jewish population, thus supporting the pathogenicity of this variant independently from the association with another pathogenic variant [30].

In cell-based assays, overexpression of the protein might in principle mask or attenuate differences in ion transport function. Therefore, we normally apply additional tests to better characterize the molecular phenotype of a given variant [28,29]. Colocalization of human pendrin p.L117F with the plasma membrane failed to reveal differences with the wild type (Figure 3). On the other hand, good targeting of the protein to the plasma membrane was consistent with a substantial residual ion transport activity (Figure 1a).

The determination of the protein levels strikingly revealed a marked difference between the wild type and p.L117F (Figure 2b–e). Reduction in protein levels, associated with a lack of reduction in transcript levels (Figure 2a), is indicative of an accelerated degradation of a partly misfolded, dysfunctional protein. Accordingly, colocalization with the endoplasmic reticulum revealed a partial but significant retention of human pendrin p.L117F in the subcellular compartments (Figure 4).

According to the HL-EP specifications of ACMG/AMP guidelines, a variant-specific knock-in mouse model that demonstrates the phenotype is a strong indication of pathogenicity (pathogenic strong criterion PS3) [15,16]. However, mice homozygous for the East Asian population founder mutation p.H723R, as well as mice homozygous for p.T721M or p.C565Y, showed normal audiovestibular phenotypes and inner ear morphology [46,47,48]. These results show that variants that are pathogenic in humans can be non-pathogenic in mice and that differences in function between pendrin human and mouse variants are possible.

To ascertain whether a mouse carrying the p.L117F variant could represent a good model for pendrin-linked hearing loss, we characterized the ion transport function as well as expression levels of mouse pendrin p.L117F. The ion transport features of wild type mouse and human pendrin appeared to be identical in our fluorescence-based functional test (Figure 1). Similarly to the human ortholog, mouse p.L117F showed a moderated (22%) but significant reduction in ion transport efficiency compared to the wild type (Figure 1b). Likewise, mouse p.L117F also showed a significant reduction in protein levels compared to the wild type (Figure 5a). Therefore, the main functional molecular features of human pendrin p.L117F are well recapitulated by the mouse ortholog.

The exposure of cells to the proteasome inhibitor carfilzomib increased the expression of mouse pendrin p.L117F above the levels of the wild type, thus showing that the reduction of protein levels of this variant is a consequence of an accelerated degradation by the UPS (Figure 5a). Importantly, the inhibition of UPS could restore the ion transport efficiency of mouse pendrin p.L117F to the levels of the wild type (Figure 5b), suggesting that the targeting of UPS might represent an effective strategy to rescue the function of hypofunctional pendrin protein variants.

## 5. Conclusions

A triad of cell-based assays consisting of measurements of the ion transport activity by a fluorometric method, determination of the subcellular localization, and assessment of protein expression levels consistently support the pathogenicity of the Ashkenazi Jewish founder variant *SLC26A4* NM_000441.1:c.349C>T, p.L117F. The functional and molecular defects of human pendrin p.L117F were mirrored by mouse pendrin p.L117F, thus indicating that a mouse model carrying this variant might well recapitulate the pathological features of Pendred syndrome/DFNB4. Protein levels of human and mouse pendrin p.L117F were reduced compared to the wild type as a consequence of an accelerated degradation by the UPS. The determination of protein levels might represent a useful tool to disclose molecular defects masked by a modest reduction in ion transport function and help discriminate between pathogenic and non-pathogenic pendrin protein variants.

## Data Availability

The data that support the findings of this study are available from the corresponding author upon reasonable request.
